# Genome‐wide DNA methylation analysis by MethylRad and the transcriptome profiles reveal the potential cancer‐related lncRNAs in colon cancer

**DOI:** 10.1002/cam4.3412

**Published:** 2020-09-01

**Authors:** Guixi Zheng, Yuzhi Zhang, Hongchun Wang, E Ding, Ailin Qu, Peng Su, Yongmei Yang, Mingjin Zou, Yi Zhang

**Affiliations:** ^1^ Department of Clinical Laboratory Qilu Hospital of Shandong University Jinan Shandong China; ^2^ Department of Clinical Laboratory Affiliated Hospital of Weifang Medical University Weifang Shandong China; ^3^ Department of Pathology Shandong University School of Medicine Jinan Shandong China

**Keywords:** Bioinformatic analysis, Colon cancer, lncRNA gene methylation, MethylRAD

## Abstract

Colon cancer (CC) is characterized by global aberrant DNA methylation that may affect gene expression and genomic stability. A series of studies have demonstrated that DNA methylation could regulate the expressions of not only protein‐coding genes but also ncRNAs. However, the regulatory role of lncRNA genes methylaton in CC remains largely unknown. In the present study, we systemically characterize the profile of DNA methylation, especially the aberrant methylation of lncRNAs genes using MethylRAD technology. A total of 132 999 CCGG/8487 CCWGG sites were identified as differentially methylated sites (DMSs), which were mainly located on the introns and intergenic elements. Moreover, 1,359 CCGG/1,052 CCWGG differentially methylated genes (DMGs) were screened. Our results demonstrated that aberrant methylation of lncRNA genes occurred most frequently, accounting for 37.5% and 44.3% in CCGG and CCWGG DMGs respectively. In addition, 963 lncRNA DMGs were co‐analyzed with 1328 differentially expressed lncRNAs which were identified from TCGA database. We found that 15 lncRNAs might be CC‐related lncRNAs. ZNF667‐AS1 and MAFA‐AS1 were down‐regulated in CC, which might be silenced by hypermethylation. Besides, 13 lncRNAs were hypomethylated and up‐regulated in CC. Moreover, our results validated the expression and methylation level of CC‐related lncRNAs by RT‐qPCR and pyrosequencing assay. In conclusion, we performed a genome‐wide DNA methylation analysis by MethylRAD to acquire both CCGG and CCWGG DMSs and DMGs in CC. The results screened lncRNA DMSs as potential biomarkers and identified 15 lncRNAs as CC‐related lncRNAs. This study provided novel therapy targets and valuable insights into molecular mechanism in tumorigenesis and development of CC.

## Graphical abstract

We performed a genome‐wide DNA methylation analysis by MethylRAD to acquire both CCGG and CCWGG DMSs and DMGs in CC. Our results screened lncRNA DMSs as potential biomarkers and identified 15 lncRNAs as CC‐related lncRNAs. This study provided novel therapy targets and valuable insights into molecular mechanism in tumorigenesis and development of CC.

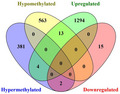

## 1 INTRODUCTION

Colorectal cancer (CRC) is the third most common malignant cancer worldwide, and it is also the second leading cause of cancer‐related deaths. The World Health Organization (WHO) predicts a substantial increase of newly diagnosed CRC cases and an increase of 80% in CRC‐related deaths by 2030.^1^ Colon cancer (CC) and rectal cancer (RC) are usually recognized as a single tumor entity called CRC in all fields of research and clinical practice. However, there are obvious differences in molecular mechanism, pathology, surgical topography, and multimodal treatment between CC and RC.^2^ In the present study, we focused on CC which is resulted from the accumulation of a variety of genetic and epigenetic alterations in colon epithelial cells.

Several studies have demonstrated that epigenetic alterations occur earlier and more frequently than genetic alterations in CC.^3^, ^4^ Approximately 75% CC arises from long‐term accumulation of epigenetic alterations.^5^ In the past decades, a large number of epigenetic aberrations have been identified in CC patients, such as DNA methylation, histone modification, and expressions of microRNAs and long non‐coding RNAs (lncRNAs).^6^ Global DNA hypomethylation has been considered as a common epigenetic feature in CC patients. Some DNA methylations, such as SEPT9, MLH1, APC, LINE‐1, and so on, have been identified as markers for early detection, prognosis and/or treatment response of CC.^7^, ^8^ It has also been reported that silencing of multiple tumor suppressor genes by DNA hypermethylation plays a key role in the process of initiation, development, and metastasis of CC.^9^

Previous studies on regulation of DNA methylation mainly focus on protein‐coding genes. Protein‐coding genes are less than 2% of the whole genome, and more than 80% genes are transcribed into noncoding RNAs (ncRNAs). A series of studies have demonstrated that DNA methylation not only regulates the expressions of protein‐coding genes but also affects ncRNAs (including miRNAs and lncRNAs).^10^, ^11^ ncRNAs have attracted immense research attention, and quite a few miRNAs and lncRNAs are dysregulated and involved in the initiation and progression of CC.^9^ However, the regulatory role of methylaton of lncRNA genes in CC remains largely unknown.

In the present study, we aimed to identify cancer‐related lncRNAs in CC by co‐analysis of methylation and transcriptome profiles. Firstly, we conducted the genome‐wide DNA methylation profiling of five paired CC tumor tissues and adjacent normal tissues using MethylRAD technology, followed by high‐throughput sequencing. We screened the differentially methylated sites (DMSs) and differentially methylated genes (DMGs) of both CCGG and CCWGG. Moreover, the RNA sequencing data of CC patients were downloaded from The Cancer Genome Atlas (TCGA) database, and the differentially expressed lncRNAs were analyzed. We found that the dysregulated lncRNAs might be caused by aberrant methylation. Finally, we identified that ZNF667‐AS1 and MAFA‐AS1 were hypermethylated and down‐regulated, while the other 13 lncRNAs were hypomethylated and up‐regulated in CC. These findings suggested that those above‐mentioned 15 lncRNAs were CC‐related lncRNAs. Furthermore, we validated the expression and methylation levels of these 15 lncRNAs by RT‐qPCR and pyrosequencing.

## 2 MATERIAL AND METHODS

### 2.1 Subjects

Five CC patients for MethylRad assay and 20 CC patients for validation assay were recruited from the Department of General Surgery, Qilu Hospital of Shandong University, between December 2013 and December 2016. The diagnosis of CC was confirmed by histopathology or histobiopsy. CC tissues and paired adjacent normal mucosal tissues were collected since CC is a malignant epithelial tumor and originate from glandular epithelium of the colonic mucosa. All tissues were immediately frozen in liquid nitrogen and stored at −80°C prior to DNA extraction. Written informed consent was obtained from every participant for use of tissue samples.

### 2.2 MethylRAD library preparation and sequencing

The MethylRAD libraries were constructed using a previously described protocol.^12^ Briefly, 200 ng DNA was digested with enzyme FspEI (NEB), and the adapters were ligated to the DNA fragments. Subsequently, ligation products were amplified by PCR, and the resultant amplicons were purified using QIAquick PCR Purification Kit (Qiagen) and then pooled for single‐end sequencing using the Illumina HiseqXTen platform.

Raw reads were first subjected to quality filtering and adaptor trimming. Base quality values were calculated using a Phred quality score (Qsanger=−10log10p). The high‐quality (HQ) reads were subsequently aligned against the genome sequence of *Crassostrea gigas*. FspEI sites (CmC/mCDS) extracted from the genome of *Crassostrea gigas* were built as reference sites, and HQ reads were mapped against these reference sites using the SOAP program. For relative quantification of MethylRAD data, the number of reads supporting methylcytosines was calculated and normalized as RPM (reads per million).

### 2.3 Raw data re‐filtering and quality control

The sequences containing adapters and low‐quality reads were excluded from raw reads, and clean reads were generated. Pear software was used to re‐filter paired clean reads according to the two criteria as follows: (a) low‐quality reads containing more than 15% of bases, of which the quality scores were lower than 30, were excluded, and (b) sequences containing more than 8% N base were excluded. Then the enzyme reads of each sample were generated by deleting the labels without the digestion site of FspEI enzyme.

### 2.4 Identification of DMSs

The labels containing CCGG or CCWGG sites were extracted as reference sequences. Then the enzyme reads were mapped to the established reference sequences using SOAP software. Moreover, the enzyme reads with the unique alignment position on the reference gene were considered as mapping reads. The methylation levels of CCGG and CCWGG sites were reflected by the sequencing depth of the methylation label. The relative quantification value of methylation level of sites in each sample was calculated by site coverage reads/HQ read number of the library*10^6^. The DMSs between tumor and control groups were analyzed by EdgeR using R software. The methylated sites with *P* < .05 and log_2_FC > 1 between the two groups were considered as DMSs.

### 2.5 Identification of DMGs

The methylation level of each gene was represented by the sum of methylation level of all the methylated sites in this gene. The differential methylation level of gene was compared between tumor and adjacent normal tissue groups. The methylated genes with *P* < .05 and log_2_FC > 1 were considered as DMGs.

### 2.6 The annotation of lncRNAs

The data annotation of human lncRNAs was retrieved from GENCODE v25. The gene name, sequence and description were retrieved from HGNC using R script. The lncRNA DMSs and DMGs were selected after annotation.

### 2.7 Identification of differentially expressed lncRNAs in TCGA database

The RNA sequencing data in CC was downloaded from TCGA database using R script. The differential expression level of each gene was analyzed by DESeq using R script. The lncRNAs with *P* < .05 and log_2_FC > 1 were considered as differentially expressed lncRNAs.

### 2.8 Identification of CC‐related lncRNAs

The lncRNA DMGs in methylation file and dysregulated lncRNAs in RNA sequencing file were co‐analyzed by Venny 2.1. The lncRNAs, which were hypermethylated and down‐regulated, or were hypomethylated and up‐regulated in CC, were screened to perform further functional analysis.

### 2.9 The functional enrichment analysis of cancer‐related lncRNAs

LncRNA2Target v2.0 and StarBase v3.0 were used to predict the functions of cancer‐related lncRNAs.^13^, ^14^ LncRNA2Target v2.0 provides a comprehensive resource of lncRNA‐target relationships, which were validated by immunoprecipitation assay, RNA pull‐down assay and luciferase reporter assay. StarBase v3.0 is an open‐source platform for studying the miRNA‐ncRNA, miRNA‐mRNA, ncRNA‐RNA, RNA‐RNA, RBP‐ncRNA, and RBP‐mRNA interactions from CLIP‐Seq, degradome‐seq, and RNA‐RNA interactome data. It identifies more than 1.5 million RNA‐RNA interactions from multidimensional sequencing data. The co‐expressed protein‐coding genes were screened for Gene Ontology (GO) enrichment analysis and Kyoto Encyclopedia of Genes and Genomes (KEGG) pathway analysis. The GO categories and enriched pathways were reported as significant only if the *P* value < .05.

### 2.10 The prediction of lncRNA‐miRNA interactions

DIANA‐LncBase experimental v.2 was employed to predict miRNAs that interacted with cancer‐related lncRNAs.^15^ DIANA‐Tools provides experimentally verified miRNA targets on protein‐coding RNAs and ncRNAs (TarBase v7.0 and LncBase).

### 2.11 Quantitative real‐time qPCR

Total RNA was isolated from 20 paired tumor and adjacent nontumor tissues of CC patients using Trizol reagent (Thermo Fisher Scientific) according to the manufacturer's instructions. The cDNA was synthesized using the Prime Script™ RT Reagent Kit (Takara, Dalian, China). The reaction volume was 20 µL consisting of 1 µg of template RNA, 4 µL of 5 × Prime Script buffer mix, 1 µL of oligo dT primer, and 1 µL of Prime Script RT enzyme mix and ddH_2_O. The mixture was first incubated at 37°C for 30 minutes, followed by 85°C for 5 seconds. Then, qPCR was performed with SYBR Premix ExTaq™ (Takara) on the CFX96TM Real‐Time System (Bio‐Rad Laboratories Inc, Hercules, CA, USA). The thermocycling profile of reaction was 95°C for 5min, followed by 40 cycles of 95°C for 5s, 60°C for 20s, and 72°C 20s. The relative expression of lncRNAs was calculated using 2^‐ΔΔCt^ method. The primer sequences of lncRNAs are shown in Table S1.

### 2.12 Pyrosequencing assay

Genomic DNA was extracted from 20 paired tumor and adjacent nontumor tissues of CC patients using the QIAamp DNA Mini Kit (Qiagen, Hilden, Germany). Then DNA was bisulfite‐converted using the EpiTect Bisulfite Kit (Qiagen, Hilden, Germany) and PCR was performed using PyroMark PCR Kit (Qiagen, Hilden, Germany) according to the manufacturer's instructions. The methylation levels of 15 lncRNAs gene were measured by pyrosequencing assay which was performed on the PyroMark Gold Q96 ID (Qiagen, Hilden, Germany). The methylation sites were selected based on our high sequencing results.

### 2.13 Statistical analysis

The expression of lncRNAs was presented as mean ± SEM of three replicate measurements in RT‐qPCR assay. Two‐tailed t‐test was used to determine statistical difference between paired tumor and adjacent nontumor tissue. The statistical difference of methylation level of lncRNA genes was assessed with t‐test between tumor group and adjacent non‐tumor group. A two‐sided *P* < .05 was considered as statistical significance. Data were analyzed using GraphPad Prism version 5.0 (GraphPad Software).

## 3 RESULTS

### 3.1 MethylRAD sequencing data re‐filtering and quality control

In the present study, we used a recently discovered DNA methylation profiling method called MethylRAD.^12^ MethylRAD uses one of the Mrr‐like enzymes FspEI to perform reduced methylome sequencing and collect 32‐bp methylated DNA fragments from the whole genome for high‐throughput sequencing. This new method can discriminate between CG and non‐CG methylations and analyze both CCGG and CCWGG methylation sites. After the raw data were re‐filtered according to the criteria mentioned in section of Materials and methods and the paired‐end sequencing reads were mapped, the clean reads, enzyme reads, and mapping reads were generated in each sample as shown in Table 1.

**Table 1:** Sequencing clean reads and re‐filtered reads in each sample

Sample	Clean Reads	Enzyme Reads	Mapping Reads	Ratio
107C	194 689 147	51 921 267	39 585 629	76.24%
107T	194 689 147	58 815 935	44 228 641	75.20%
111C	194 689 147	59 216 156	44 285 081	74.79%
111T	194 689 147	52 735 852	36 441 996	69.11%
120C	148 088 447	35 816 754	27 341 986	76.34%
120T	148 088 447	38 880 006	29 361 131	75.52%
124C	148 088 447	38 840 014	29 184 649	75.14%
124T	148 088 447	36 728 612	27 133 927	73.88%
153C	118 096 064	32 884 141	25 144 380	76.47%
153T	118 096 064	37 680 049	28 305 940	75.12%

Notes:

Adjacent tissue group:107C, 111C, 120C, 124C and 153C; Colon cancer tissue group:107T, 111T, 120T, 124T and 153T; Ratio:Mapping reads/Enzyme reads.

### 3.2 Distribution of DNA methylation sites on different functional elements and in different gene regions

**Figure 1:**
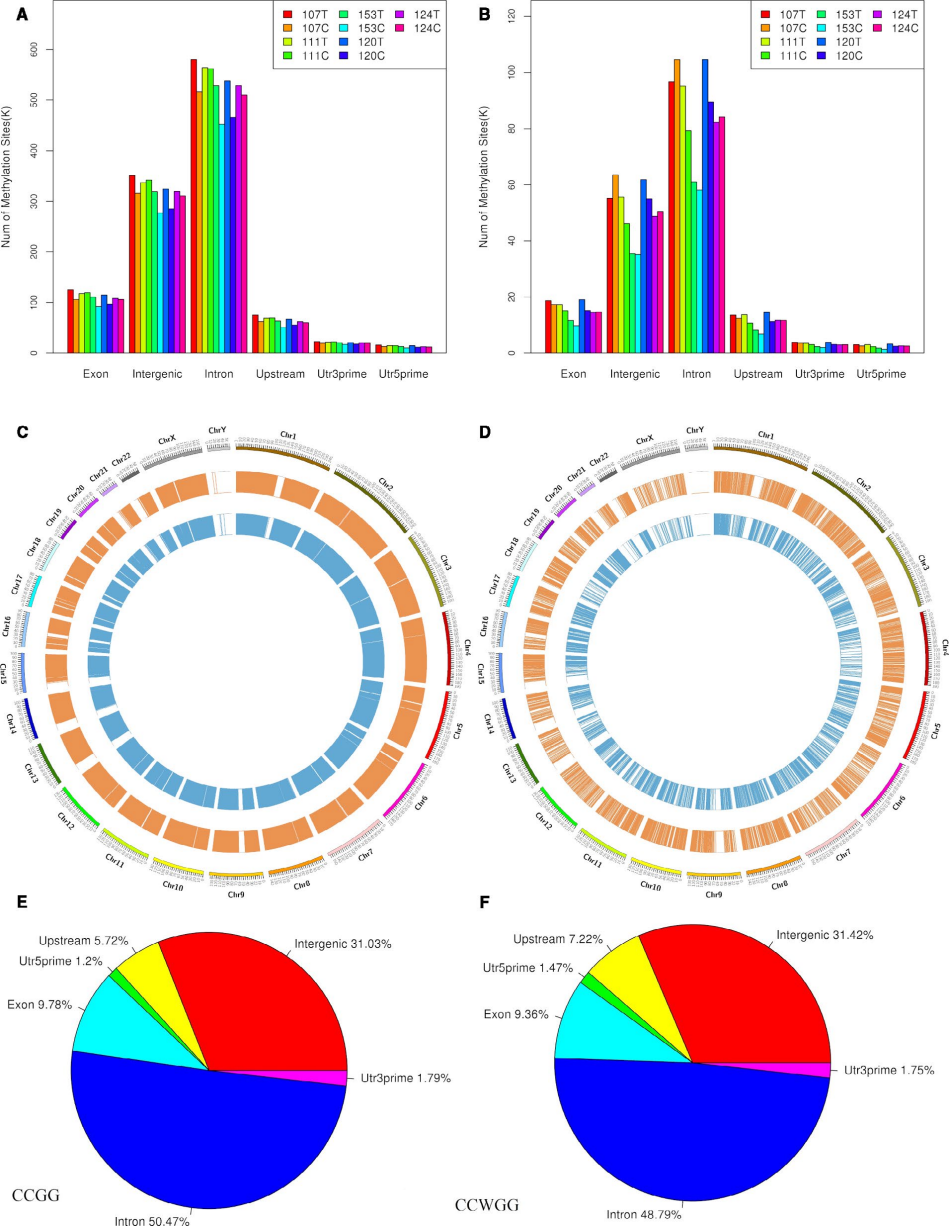
Distribution of differential methylation sites. (A) CCGG methylation sites on different gene function elements, (B) CCWGG methylation sites on different gene function elements, (C) CCGG methylation sites on different chromosomes, (D) CCWGG methylation sites on different chromosomes, (E) CCGG methylation sites on different functional components, (F) CCWGG methylation sites on different functional components

The labels containing CCGG or CCWGG sites were extracted from the whole genome sequence as reference sequences. The enzyme reads of each sample were mapped to the established reference sequences, and only the sites with sequencing depth higher than three were considered as reliable methylation sites. Figure 1A and Figure 1B illustrate that the distribution of methylation sites on different gene functional elements in each sample was analyzed by bedtools software. The results indicated that both CCGG and CCWGG methylation sites were mainly distributed on introns and intergenic elements.

### 3.3 Relative quantification of methylation levels of sites

We detected a total of 773 956 CCGG and 59 968 CCWGG methylation sites. As described in the section of Materials and methods, the methylation level of CCGG and CCWGG sites was reflected by the sequencing depth of methylation labels. The PRM was used to compare methylation level of each site between tumor and adjacent normal tissue groups. Finally, a total of 132 999 CCGG/8487 CCWGG sites were identified as DMSs.

Usually, DMSs are unevenly distributed on chromosomes. Figure 1C and Figure 1D exhibit that the position information of DMSs on chromosomes was labeled. According to the position of methylation sites on the genome, the distribution of DMSs on different functional elements was plotted using pie chart as shown in Figure 1E and Figure 1F. The results showed that both CCGG and CCWGG DMSs were mainly located on the introns and intergenic elements.

### 3.4 Identification of lncRNA DMSs

**Figure 2:**
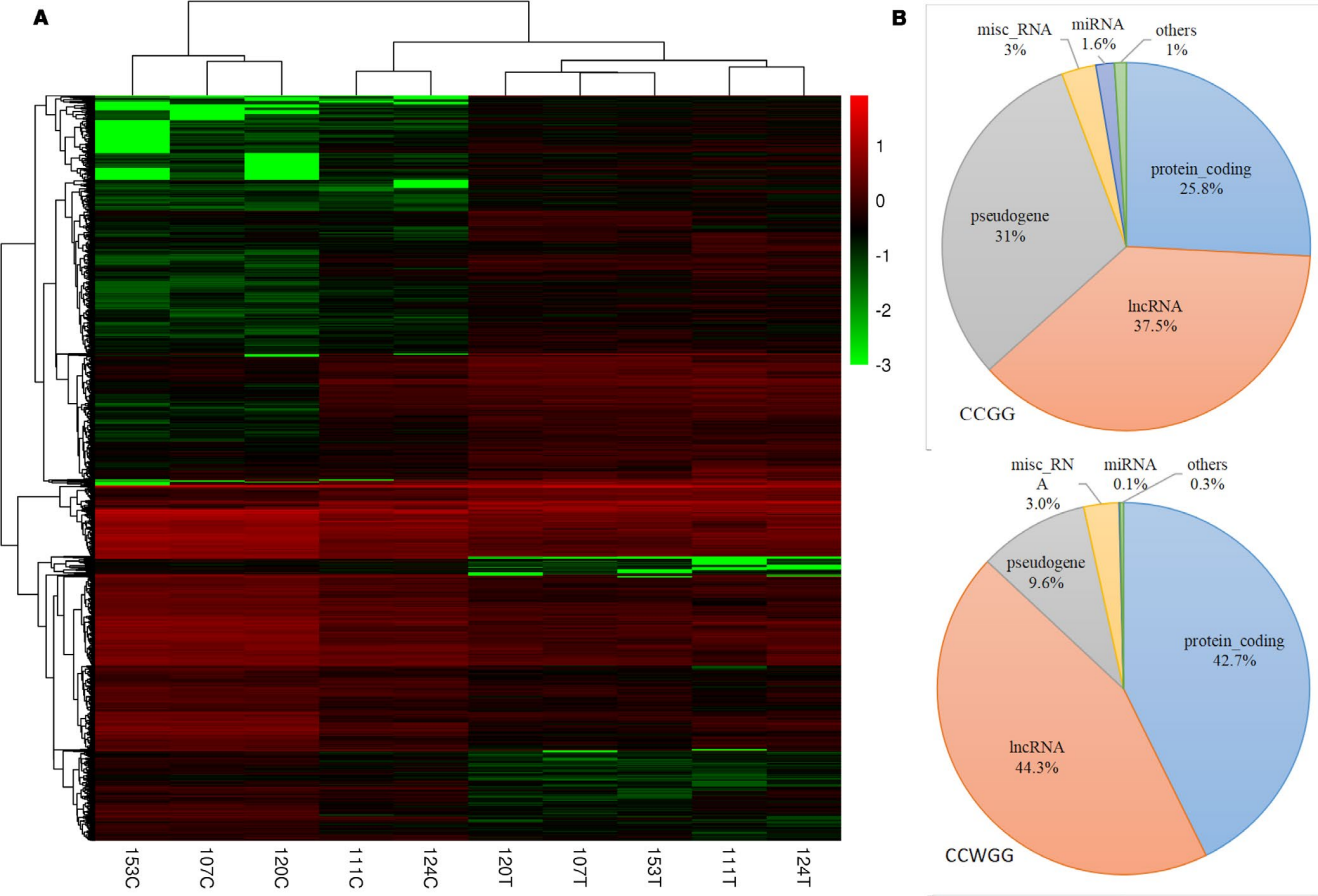
Hierarchical cluster analysis and classification of DMGs. (A) Heat‐map of top 10 000 lncRNA differentially methylated sites between tumor tissue and adjacent tissue group. (B) Classification of DMGs and the rate distribution of 6 different biotypes genes

According to the annotation data of lncRNAs retrieved from GENCODE, there were 42 175 CCGG/2794 CCWGG DMSs which belonged to lncRNA category. Finally, we obtained 44 877 lncRNA DMSs after combining CCGG and CCWGG files with the exclusion of 92 repeated DMSs. The cluster analysis was performed to further explore the differential methylation level of lncRNA DMSs. The top 10 000 lncRNA DMSs were selected to plot cluster heatmap. The heatmap plot showed a different methylation pattern between tumor tissues (T) and adjacent nontumor tissues (C). There were more DMSs with hypermethylation in tumor tissues. LncRNA DMSs could separate tumor and adjacent normal tissue groups very well as shown in Figure 2A. All the five paired of tumor tissues and adjacent nontumor tissues could be separated correctly.

### 3.5 Identification of lncRNA DMGs

The methylation level of each gene was represented by the sum of methylation levels of all the sites in this gene. According to the criteria described in the section of Materials and methods, we identified 1,359 CCGG and 1,052 CCWGG DMGs. These DMGs were further reclassified into the following biotypes: protein‐coding genes, lncRNA genes, miRNA genes, pseudogenes, misc_RNA genes, and others. Figure 2B reveals that there were 510 lncRNA genes (37.5%) and 421 pseudogenes (31%) in CCGG DMGs, and 466 lncRNA genes (44.3%) and 449 protein‐coding genes (42.7%) in CCWGG DMGs. The results demonstrated that lncRNA genes occurred frequently in both CCGG and CCWGG DMGs. Finally, we identified 963 lncRNA DMGs (Table S2) for further co‐analysis with TCGA RNA sequencing data.

### 3.6 Identification of differentially expressed lncRNAs in TCGA database

The RNA sequencing data of 480 CC patients and 41 controls were downloaded from TCGA database using R script. A total of 3693 differentially expressed genes (DEGs) were obtained by DEseq. According to Ensemble Genes 95 and Human genes GRCh38.p12 database, there were 1,328 DEGs in lncRNA category (Table S3).

### 3.7 Identification of CC‐related lncRNAs

**Figure 3:**
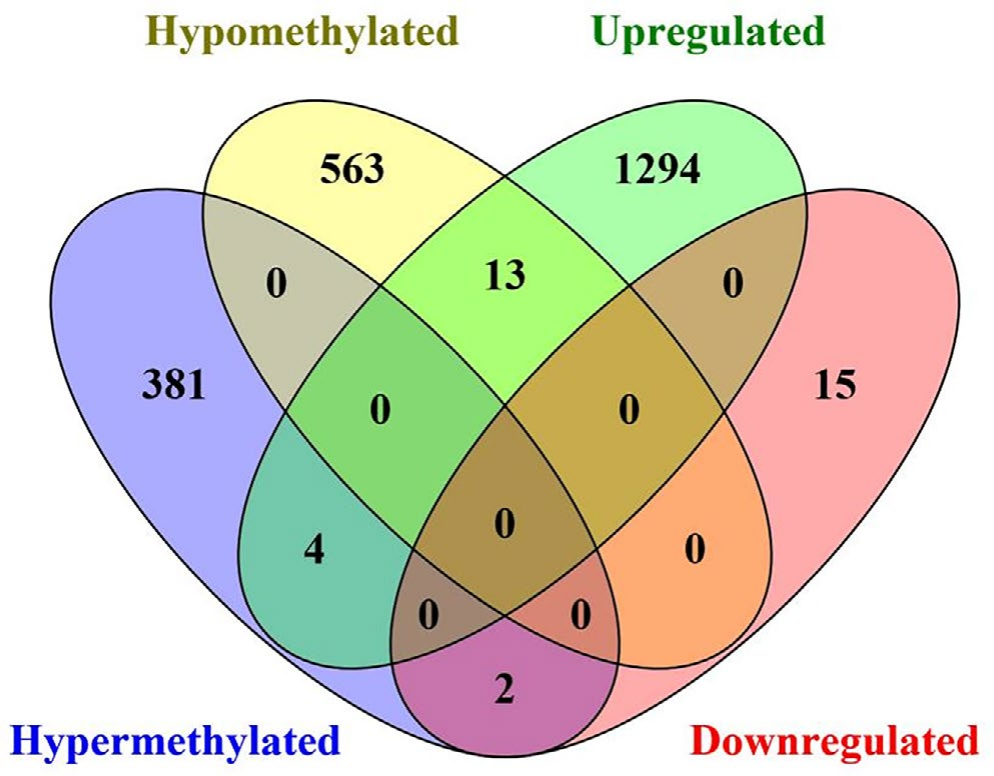
Venny plot of lncRNA differentially methylated gens (DMGs) in MethylRad sequencing data and differentially expressed lncRNAs in TCGA database

The lncRNA DMGs and dysregulated lncRNAs were co‐analyzed by Venny 2.1. There were 963 lncRNA DMGs, including 387 hypermethylated and 576 hypomethylated ones, and 1328 dysregulated lncRNAs, including 1,311 up‐regulated and 17 down‐regulated lncRNAs. Finally, we identified that ZNF667‐AS1 and MAFA‐AS1, which was down‐regulated, might be silenced by hypermethylation. Moreover, the other 13 lncRNAs were hypomethylated and up‐regulated in CC (Figure 3, Table 2). Our result demonstrated that these 15 lncRNAs might be CC‐related lncRNAs.

**Table 2:** The list of colon cancer‐related lncRNAs

Gene ID	Ensembl_gene_ID	Gene Name	Methylation level		expression level	
			log_2_FC	*P* value	log_2_FC	*P* value
100 128 252	ENSG00000166770	ZNF667‐AS1	3.181057633	3.12E‐09	‐1.3072	.01356
104 326 051	ENSG00000254338	MAFA‐AS1	1.034955058	.008881073	‐1.150617756	.023423169
100 505 658	ENSG00000246422	AC008781.2	‐2.053666981	.0000128	2.254499463	.001472013
728 655	ENSG00000251164	HULC	‐1.376854024	.000863543	2.512989215	5.59E‐05
105 370 855	ENSG00000259756	AC100839.1	‐1.361977359	.009511436	2.784632131	.000976912
101 926 928	ENSG00000258551	CRAT37	‐1.349790211	.001218479	1.212392239	.011940946
101 929 344	ENSG00000231817	LINC01198	‐1.325550476	.00542864	1.932731982	.001108353
101 928 104	ENSG00000267659	LINC01482	‐1.301990851	.001205346	1.384972804	.007679533
101 927 659	ENSG00000250582	SMAD1‐AS2	‐1.255865686	.008947149	2.900569484	.006680646
101 927 761	ENSG00000223442	TH2LCRR	‐1.201145929	.002381	1.086872425	.000338205
104 472 714	ENSG00000226758	DISC1‐IT1	‐1.170968744	.002726115	1.944068824	.036600097
100 873 982	ENSG00000223882	ABCC5‐AS1	‐1.15089644	.003446008	2.079893143	.004943804
645 030	ENSG00000237853	NFIA‐AS1	‐1.028709419	.002193182	3.888287973	.0000251
101 927 539	ENSG00000263499	AC007431.1	‐1.005971442	.011993399	3.879714114	.00000419
101 927 948	ENSG00000229494	AC012494.1	‐1.002646894	.000000206	2.146082342	.003494868

### 3.8 The functional enrichment analysis of cancer‐related lncRNAs

To explore the potential functions of these cancer‐related lncRNAs, we attempted to identify the protein‐coding genes that were co‐expressed with these lncRNAs using LncRN2Target v2.0 and StarBase. Among the input set of 15 cancer‐related lncRNAs, 28 and nine co‐expressed protein‐coding genes were identified to be associated with HULC and ZNF667‐AS1, respectively (Table 3).

**Table 3:** Co‐expressed protein‐coding genes of lncRNA ZNF667‐AS1 and HULC

LncRNA	Co‐expressed protein‐coding genes	Database
ZNF667‐AS1	CALCOCO2, FUNDC2, HMGCS1, CAPRIN1, PAFAH1B2, ZNF814, AC010326.2, EIF3C, PPP1CC	Starbase
HULC	ANKRD11, CDCA4, CDK8, CEP83, CWF19L1, GGT1, IWS1, LTBR, MKNK2, MORC2, NDUFC2, NNT, NOP53, NUCKS1, RPRD1A, SDC4, SH3D19, SNAPC2, TBCB, TM9SF3, VPS4A, ZNF195, CDKN2C, UBE2I, E2F1, EZH2, PTGS2, HMGA2	LncRNA2tARGET v2.0 Starbase

### 3.9 Prediction of lncRNA‐miRNA interaction

According to DIANA‐LncBase experimental v.2 results, we found that six miRNAs and seven miRNAs could bind to lncRNA ZNF667‐AS1 and HULC respectively. These lncRN‐miRNA interactions were verified by immunoprecipation, reporter gene assay or qPCR analysis (Table 4).

**Table 4:** The miRNAs that were experimentally proved to bind lncRNA ZNF667‐AS1 and HULC

LncRNAs	miRNAs	PrScore	Methods
ZNF667‐AS1	hsa‐miR‐574‐5p^34^	0.404	IP
	hsa‐miR‐143‐3p^34^	0.658	
	hsa‐miR‐330‐3p^35^	—	
	hsa‐miR‐33a‐3p^34^	—	
	hsa‐miR‐484^34^	—	
	hsa‐miR‐1‐3p^34^	0.586	
HULC	hsa‐miR‐1236‐3p^36^	0.468	RP
	hsa‐miR‐134‐5p^36^	—	RP
	hsa‐miR‐372‐3p^36^	—	RP,qP
	hsa‐miR‐433‐3p^36^	0.425	RP
	hsa‐miR‐557^36^	—	RP
	hsa‐miR‐613^36^	—	RP,qP
	hsa‐miR‐622^36^	—	RP

Notes:

The data of IP, RP, qPCR was obtained from previous studies as cited references.

Abbreviations: IP: positive by immunoprecipitation; RP: positive by reporter gene assay; qP: positive by qPCR.

### 3.10 Validation of the expression and methylation level of CC‐related lncRNAs

**Figure 4:**
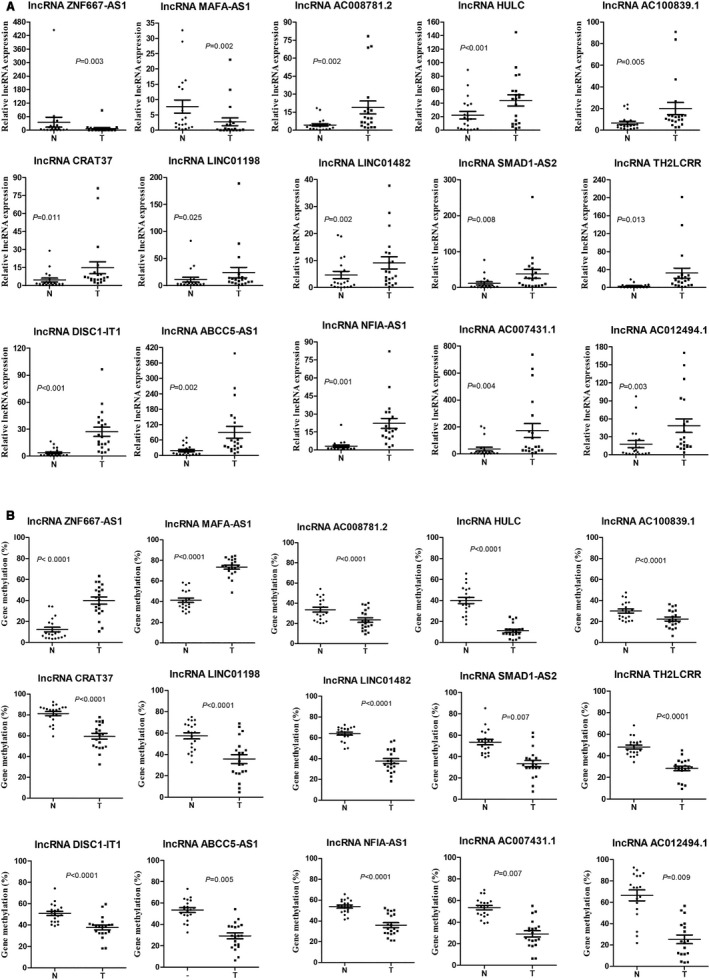
The expressions of CC‐related lncRNAs by RT‐qPCR (A) and aberrant methylation levels of the genes by pyrosequencing (B) in 20 cases of CC tumor and non‐tumor tissues. RT‐qPCR data are presented as mean ± SEM of three replicate measurements. Statistical significance of the differences is assessed by two‐tailed t‐test between tumor group and adjacent nontumor group

In order to validate our MethylRad sequencing results and TCGA data, the expression and methylation levels of 15 CC‐related lncRNAs were detected by RT‐qPCR and pyrosequencing. Our results indicated that lncRNA ZNF667‐AS1 and MAFA‐AS1 were down‐regulated and hypermethylated in CC tumor tissues (Figure 4A and B). Meanwhile, the other 13 lncRNAs (AC008781.2, HULC, AC100839.1, CRAT37, LINC01198, LINC01482, SMAD1‐AS2, TH2LCRR, DISC1‐IT1, ABCC5‐AS1, NFIA‐AS1, AC007431.1, AC012494.1) were up‐regulated and hypomethylated in CC tumor group which confirmed our previous MethylRad sequencing results (Figure 4A and B).

## 4 DISCUSSION

Aberrant DNA methylation has been shown to play an important role in the pathogenesis of CC. Previous studies have identified that methylation changes are associated with a limited set of genes in CC.^16^, ^17^, ^18^, ^19^ However, the profiles of DNA methylation, especially the menthylation of lncRNA genes in CC, still remain largely unexplored. With rapid advances in sequencing technology, sequencing‐based methods have been promising in identifying DNA methylation sites and genes on a genome‐wide scale.

In the present study, we used a simple and scalable DNA methylation profiling method called MethylRad, which can collect 32‐bp methylated DNA fragments from the whole genome for further high‐throughput sequencing. MethylRAD can discriminate between CG and non‐CG methylations, and analyze both CCGG and CCWGG methylation sites, whereas most of other methods can either only recognize CG methylation or recognize both CG and non‐CG methylations but cannot distinguish them.^12^, ^20^ We detected a total of 773 956 CCGG and 59 968 CCWGG sites by generating a genome‐wide DNA methylation profile using five pairs of CC tumor tissues and adjacent normal tissues. We also analyzed the distribution of DNA methylation sites on different functional elements and in different gene regions. Our results indicated that CCGG and CCWGG methylated sites were mainly distributed on introns and intergenic elements.

Moreover, a total of 132 999 CCGG and 8487 CCWGG sites were identified as DMSs, which was consistent with the previous studies that there is an increasing number of hypermethylated CGIs in CC.^21^ According to the annotation data of lncRNAs retrieved from GENCODE, there were 44 877 lncRNA DMSs after combining CCGG and CCWGG files. The top 10 000 lncRNA DMSs were selected to plot cluster heatmap of tumor and adjacent normal tissue groups. The results demonstrated that the lncRNA DMSs could separate tumor and adjacent normal tissue groups and might provide potential tumor markers of CC. The methylation levels of these lncRNA DMSs need to be verified by pyrosequencing in the future research.

In order to explore the candidate differentially methylated lncRNA genes that might contribute to tumorigenesis and development of CC, we identified 1359 CCGG and 1052 CCWGG DMGs. These DMGs mainly belonged to lncRNA genes and pseudogenes in CCGG DMGs, while they were mainly lncRNA genes and protein‐coding genes in CCWGG DMGs. Our results were consistent with previous studies that DNA methylation not only regulates the expressions of protein‐coding genes but also affect ncRNAs. Li et al have also reported that aberrant methylation changes occur more frequently in the promoters of ncRNA genes compared with protein‐coding genes in breast cancer.^10^

To better understand the potential effect of methylation on lncRNA expression, the RNA sequencing data of CC patients were downloaded from TCGA database and analyzed. We identified 1328 DEGs in lncRNA category. The lncRNA DMGs and dysregulated lncRNAs were co‐analyzed to identify cancer‐related lncRNAs. We identified two lncRNAs, which were down‐regulated and might be silenced by hypermethylation. In addition, the other 13 lncRNAs were hypomethylated and up‐regulated. The expression and methylation results of these 15 CC‐related lncRNAs were also validated by RT‐qPCR and pyrosequencing assay. Our results might provide new insights into tumorigenesis and development of CC.

Among the 15 cancer‐related lncRNAs, ZNF667‐AS1, HULC, LINC01198, and NFIA‐AS1 have been reported in previous studies. We also identified protein‐coding genes and microRNAs that were interacted with ZNF667‐AS1 and HULC. As far as we know, there are no studies about the interaction role of the other 13 lncRNAs. It has been demonstrated that lncRNA HULC is markedly up‐regulated in CC patients and CC cell lines.^22^ HULC accelerates the growth of CC cells by targeting miR‐613. These findings suggest that HULC inhibits the tumor growth through miR‐613‐dependent RTKN modulation.^23^ HULC may be an oncogenic lncRNA, which promotes the progression of CC and can be considered as an effective therapeutic target in human CC.^24^ Our study also found that HULC was hypomenthylated and up‐regulated in CC, which was consistent with previous studies. Vrba et al have reported that ZNF667‐AS1 expression is silenced by aberrant DNA methylation in several cancers, providing strong evidence for a suppressor role of ZNF667‐AS1.^25^ Several studies have also demonstrated that ZNF667‐AS1 is down‐regulated and may serve as a tumor suppressor gene in breast and cervical cancer.^26^, ^27^ In our present study, we validated that ZNF667‐AS1 was hypermethylated and down‐regulated in CC patients. However, the role of ZNF667‐AS1 in CC still needs to be clarified in further investigation. Several studies have demonstrated that LINC01198 was up‐regulated in glioblastmoma and associated with clinical outcomes.^28^, ^29^, ^30^ LINC01198 could activate PI3/AKT signaling to facilitate gliomagenesis through regulating PIK3CA and PTEN.^31^ Sun et al ^32^ have found that LINC01198 was one of the high‐risk factors for bladder cancer tumors. Shao et al^33^ have indicated that NFIA‐AS1 was down‐regulated and might be a promising biomarker for ER positive breast cancer. It still needs further study about the other CC‐related lncRNAs.

Collectively, we performed a genome‐wide methylation analysis by MethylRad and transcriptome profiles from TCGA database in CC. We identified DMSs on lncRNA genes, differentially methylated lncRNA genes and also dysregulated lncRNAs. Finally, we found that ZNF667‐AS1 and MAFA‐AS1 were hypermethylated and down‐regulated, while the other 13 lncRNAs were hypomethylated and up‐regulated in CC. In contrast to the extensive evidence of protein‐coding genes, only few lncRNAs have been clearly elucidated till now. In the present study, we proposed 15 lncRNAs as potential CC‐related lncRNAs, and they might participate in the tumorigenesis and progression of cancer. Taken together, our study provided useful diagnostic markers and new insights into molecular mechanisms of CC.

## CONFLICTS OF INTEREST

The authors declared that they have no conflicts of interest.

## AUTHOR’S CONTRIBUTION

GZ carried out most of experiments and data analysis, interpreted the entire results and drafted the manuscript. YZ and ED carried out part of statistic analysis. PS confirmed the diagnosis of colon cancer. HW, YY and AQ carried out TCGA data analysis. All authors read and approved the final manuscript. YZ conceived and designed the study.

## Supporting information

Table S1

Table S2

Table S3

## Data Availability

The authors confirm that the data supportting the findings of this study are available from the corresponding author YZ on request.
